# Design and Application of p-AlGaN Short Period Superlattice

**DOI:** 10.3390/mi16080877

**Published:** 2025-07-29

**Authors:** Yang Liu, Changhao Chen, Xiaowei Zhou, Peixian Li, Bo Yang, Yongfeng Zhang, Junchun Bai

**Affiliations:** 1 School of Advanced Materials and Nanotechnology, Xidian University, Xi’an 710071, China; yliu_333@stu.xidian.edu.cn (Y.L.);; 2 State Key Discipline Laboratory of Wide Band Gap Semiconductor Technology, Xidian University, Xi’an 710071, China

**Keywords:** superlattice, periodic thickness, hole concentration, enhancement-mode, threshold voltage

## Abstract

AlGaN-based high-electron-mobility transistors are critical for next-generation power electronics and radio-frequency applications, yet achieving stable enhancement-mode operation with a high threshold voltage remains a key challenge. In this work, we designed p-AlGaN superlattices with different structures and performed energy band structure simulations using the device simulation software Silvaco. The results demonstrate that thin barrier structures lead to reduced acceptor incorporation, thereby decreasing the number of ionized acceptors, while facilitating vertical hole transport. Superlattice samples with varying periodic thicknesses were grown via metal-organic chemical vapor deposition, and their crystalline quality and electrical properties were characterized. The findings reveal that although gradient-thickness barriers contribute to enhancing hole concentration, the presence of thick barrier layers restricts hole tunneling and induces stronger scattering, ultimately increasing resistivity. In addition, we simulated the structure of the enhancement-mode HEMT with p-AlGaN as the under-gate material. Analysis of its energy band structure and channel carrier concentration indicates that adopting p-AlGaN superlattices as the under-gate material facilitates achieving a higher threshold voltage in enhancement-mode HEMT devices, which is crucial for improving device reliability and reducing power loss in practical applications such as electric vehicles.

## 1. Introduction

Gallium nitride (GaN), a representative of third-generation semiconductors, features a direct wide bandgap, high breakdown field strength, high electron saturation drift velocity, and high thermal conductivity. These properties enable its extensive applications in fields requiring high breakdown electric fields and high operating voltages. Additionally, GaN can form AlInGaN [1,2,3] ternary alloys with other III-V semiconductors such as AlN and InN, achieving continuous tunability of bandgap energy from 0.7 to 6.2 eV [4]. This tunability allows the corresponding emission wavelengths to cover the entire visible and ultraviolet (UV) spectral ranges [5,6]. Furthermore, by incorporating Group II (Mg) or Group VI (Si) elements to substitute metal sites, effective p-type or n-type doping can be realized [7,8]. This capability facilitates the design and fabrication of optoelectronic devices with specific functions [9,10,11] (light-emitting diodes (LEDs), laser diodes (LDs)) and power electronic devices such as high-electron-mobility transistors [12,13] (HEMTs). Notably, GaN-based photodetectors have recently garnered significant attention in solar-blind ultraviolet (UV) detection [14], and recent literature indicates that AlGaN-based ultraviolet photodetectors have achieved a responsivity of 1.4 × 10^3^ A/W and ultrafast response speeds (0.6/25.4 ns rise/fall time) [15].

The efficient p-type doping of GaN materials was initially achieved by H. Mano through low-energy electron beam irradiation [16]. The electron beam can break the Mg-H complex bonds formed between H (introduced during the growth process) and the dopant Mg. However, this method can only realize p-type characteristics in regions on the surface that the electron beam can penetrate, which strongly depends on the electron beam penetration depth. In 1991, Nakamura provided energy for the dissociation of Mg-H bonds via rapid thermal annealing (RTA) [17], thereby activating acceptors. Since then, researchers have invested substantial efforts in studying p-type doping of AlGaN materials, including co-doping methods [18], stepwise rapid annealing [19], δ-doping [20], superlattice doping [21], and other techniques [22,23]. In reality, as the Al content increases in AlGaN materials, p-type doping becomes more challenging. This is due to the reduced lattice constant, which makes it difficult for Mg atoms to occupy lattice sites. Additionally, the acceptor activation energy of Mg increases with the rise in Al content. Moreover, nitrogen vacancies exhibit low formation energy in AlGaN, leading to the formation of nitrogen vacancy acceptor compensation.

In wurtzite-structured GaN semiconductors, Ga atoms possess five valence electrons while N atoms have three valence electrons. Consequently, the Ga-N bonds are composed of four groups: three groups of electron pairs formed by one electron each from Ga and N, and one group of electron pairs derived from the outermost electrons of Ga atoms with opposite spin directions. Among these four Ga-N bond groups, three exhibit equivalent bond energies and bond lengths, whereas the remaining group differs in bond length and energy due to its distinct formation mechanism. This disparity in the origin of Ga-N bonds leads to the non-coincidence of positive and negative charge centers in wurtzite GaN crystals, resulting in strong polarization effects, including spontaneous polarization and piezoelectric polarization [24]. Currently, the highest-quality and most maturely applied GaN materials are those with metal-polar surfaces. For metal-polar (Al)GaN materials, the direction of spontaneous polarization is downward, with a polarization strength exceeding 0.084. Such a significant polarization strength enables the formation of a two-dimensional electron gas (2DEG) with a density exceeding 1 × 10^12^ cm^−2^ at the AlGaN/GaN heterojunction interface without additional doping [25]. Meanwhile, the 2DEG at the heterojunction interface exhibits high electron mobility and electron saturation velocity, making it highly suitable for the fabrication of high-electron-mobility transistor (HEMT) devices [26].

Conventional HEMT devices are depletion-mode devices [27], where the principle involves the polarization effect of the barrier layer generating positive charges at the bottom, inducing a two-dimensional electron gas (2DEG) in the channel [28]. The gate voltage controls the opening and closing of the channel. At zero gate voltage, free electrons exist in the channel, keeping it in an on-state. In contrast, enhancement-mode HEMTs [29] are normally-off devices, meaning they do not conduct when the gate voltage is zero. They typically adopt two structures: P-GaN gate [30] and MIS gate [31]. The P-GaN gate achieves zero-gate-voltage turn-off by introducing p-GaN under the gate, where holes deplete the 2DEG in the channel beneath the gate. The MIS gate structure effectively reduces the interface state density, improving device reliability and stability, but its fabrication process is more complex, and threshold voltage regulation is challenging. Compared with traditional P-GaN gates, using P-AlGaN as the under-gate material enables polarization modulation and band engineering through adjustment of the Al composition, allowing linear regulation of the threshold voltage to meet the requirements for diverse application scenarios.

Despite the promising potential of p-AlGaN for polarization modulation in E-mode HEMTs, its practical application is hindered by two critical challenges: (1) low Mg doping efficiency due to limited acceptor incorporation in high-Al-content AlGaN, and (2) high acceptor activation energy that restricts hole concentration, ultimately degrading device conductivity. These issues directly limit the threshold voltage tunability and reliability of E-mode HEMTs. To address this, we designed a superlattice structure using Silvaco TCAD simulations, followed by the growth of p-AlGaN superlattices with varied configurations via metal-organic chemical vapor deposition (MOCVD). The structural and material qualities of these superlattices were systematically characterized. Additionally, we simulated the impacts of different p-type gate underlayer structures on the threshold voltage and output characteristics of enhancement-mode high-electron-mobility transistor (HEMT) devices. The Al composition of the superlattice structure was characterized using ultraviolet–visible (UV–VIS) spectroscopy. The crystalline quality and structural properties of the superlattice were evaluated via high-resolution X-ray diffraction (HRXRD). The surface morphology of the material was examined using atomic force microscopy (AFM). The electrical properties of the superlattice were analyzed through Hall effect measurements.

## 2. Materials and Methods

**Figure 1:**
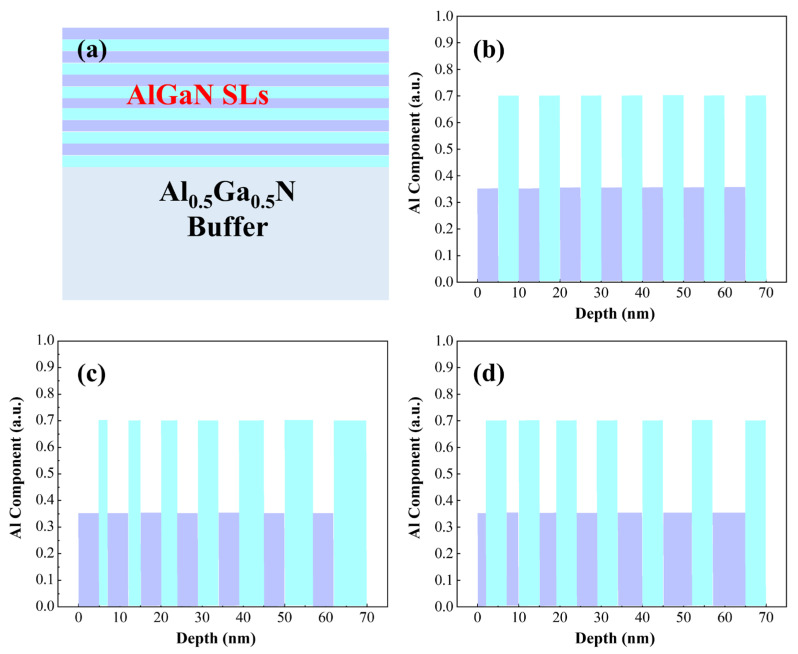
(**a**) Simulated structural diagrams of superlattice structures. (**b**) Both quantum well and barrier widths are 5 nm. (**c**) Quantum well width fixed at 5 nm with gradient barrier width. (**d**) Barrier width fixed at 5 nm with gradient quantum well width.

We designed the superlattice structures using Silvaco TCAD (2014 version) simulations. In our previous studies [32], we investigated the impacts of doping regions and Al composition in quantum wells on p-type doping in AlGaN materials, and found that doping in barrier regions combined with gradient variations in Al composition in quantum wells facilitates achieving higher hole concentrations. In this work, we designed AlGaN superlattices with different periodic configurations. The barrier layers of the superlattice structure exhibit an Al composition of 0.7, while the quantum wells show an Al composition of 0.35. Specifically, Structure A1 features both well and barrier layers with a width of 5 nm; Structure A2 maintains a fixed well width of 5 nm, with the barrier width increasing from 2 nm to 8 nm (incrementing by 1 nm per period); Structure A3 retains a fixed barrier width of 5 nm, with the well width increasing from 2 nm to 8 nm (incrementing by 1 nm per period). All samples consist of 7 superlattice periods, with a total thickness of 70 nm and an average Al composition of 0.53. The detailed superlattice configurations are illustrated in Figure 1.

**Figure 2:**
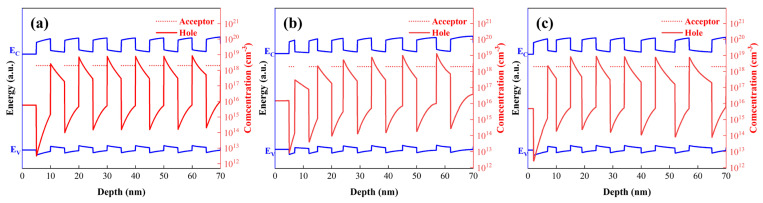
Correlation between energy band structures and hole concentrations for the three superlattice configurations. (**a**) Both quantum well and barrier widths are 5 nm. (**b**) Quantum well width fixed at 5 nm with gradient barrier width. (**c**) Barrier width fixed at 5 nm with gradient quantum well width.

As depicted in Figure 2, the energy band structures of the three superlattice configurations were simulated. Our prior work revealed that barrier layer doping is beneficial for achieving high hole concentrations. In this study, all structures were doped in the superlattice barrier layers. To ensure consistent doping levels, the doping concentration was uniformly set at 2 × 10^18^ cm^−3^ (the red dashed line in Figure 2 represents the acceptor doping concentration). Among the three structures, the highest hole concentration (~8.4 × 10^17^ cm^−3^) was obtained with the fixed barrier layer thickness configuration. The fixed well/barrier width structure yielded a hole concentration of 8.28 × 10^17^ cm^−3^. The lowest hole concentration (7.5 × 10^17^ cm^−3^) was observed in the fixed well layer thickness structure.

Notably, all samples shared identical well/barrier compositions, with only variations in well/barrier thicknesses, yet the hole density varied by over 14%. The underlying reason is that the acceptor energy levels in the barrier layers are spatially closer to the valence band maximum (VBM) of the quantum well layers, making it easier to capture electrons from the VBM of the quantum wells and leave holes behind. Thus, acceptors doped in the barrier layers tend to be activated within the quantum well regions. Due to the band bending induced by polarization effects, recombination-generated holes are prone to accumulate at the well-barrier interfaces, forming a quasi-two-dimensional hole gas (quasi-2DHG). When the barrier layer thickness is fixed and only the well layer thickness is varied, the total number of ionized acceptors in each barrier region remains nearly constant, resulting in minimal changes in the average hole concentration. Conversely, reducing the barrier layer thickness decreases the number of acceptors incorporated in this region, leading to fewer ionized acceptors (as shown by the hole concentrations in the first two well-barrier layers of Figure 2a,b). Consequently, the overall hole concentration of the superlattice structure decreases under such conditions, necessitating an increase in the barrier layer doping concentration to achieve higher hole concentrations.

**Figure 3:**
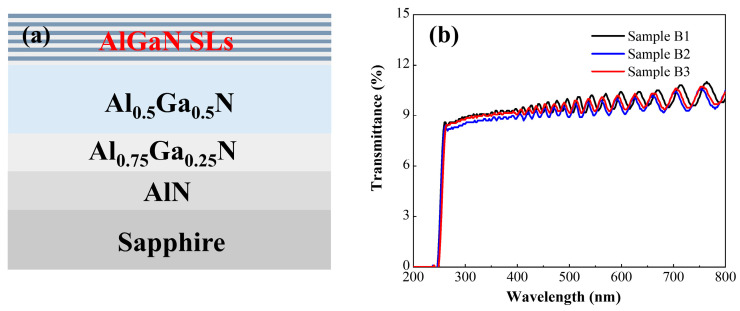
(**a**) Schematic diagram of the MOCVD-grown superlattice structure. (**b**) UV–VIS spectra of the three configurations.

To validate the simulation design, experimental growth of three superlattice structures was conducted corresponding to the three simulated configurations. Considering the challenges in growing and characterizing graded-width superlattices, the experiments were simplified. Specifically, Structure B1 features both well and barrier layers with a width of 5 nm. Structure B2 has a fixed well width of 5 nm and a barrier width of 2 nm. Structure B3 has a fixed well width of 2 nm and a barrier width of 5 nm. All samples were grown on 2-inch c-plane sapphire substrates using a metal-organic chemical vapor deposition (MOCVD) system (AIXTRON CRIUS 2, Herzogenrath, Germany). Trimethylaluminum (TMAl) and trimethylgallium (TMGa) were employed as metal sources, ammonia (NH_3_) as the nitrogen source, hydrogen (H_2_) as the carrier gas, and bis(cyclopentadienyl)magnesium (Cp_2_Mg) as the Mg dopant. As shown in Figure 3a, the buffer layer stack (from bottom to top) consists of AlN, Al_x_Ga_1−x_N, Al_0.5_Ga_0.5_N, and the superlattice structure. The average Al composition of the superlattice structure was 0.53, consistent with the well/barrier width parameters in Figure 1. The detailed growth procedures are as follows: (1) Pre-epitaxy cleaning of the sapphire substrate via heating to remove surface contaminants. (2) Surface nitridation and growth of a low-temperature AlN nucleation layer. (3) Growth of a 500-nm-thick high-temperature AlN layer. (4) Gradual adjustment of the Al composition to 0.75, followed by growth of ~500 nm Al_0.75_Ga_0.25_N. (5) Growth of a ~1 μm thick Al_0.5_GaN buffer layer. (6) Growth of the superlattice, where the period thickness was controlled by growth time. Mg doping was exclusively introduced during barrier layer growth with a Mg flow rate of 320 sccm, resulting in a 7-period superlattice. (7) Post-growth annealing of the samples in a N_2_ atmosphere.

The structure and epitaxial quality of superlattices have a strong impact on their performance, and HRXRD is a key technique for non-destructive characterization of epitaxial structures. HRXRD can verify the key parameters of superlattice design, evaluate the influence of crystalline quality on performance, and calculate the periodic thickness through satellite peak spacing to ensure matching with MOCVD growth parameters. The following section is added to the manuscript and marked in red font.

The three sample groups are completely identical except for their superlattice regions, with three superlattice structures (B1, B2, B3) grown according to the configurations in Figure 1. Structure B1 features both quantum well and barrier layers with a width of 5 nm. Structure B2 has a fixed quantum well width of 5 nm, with the barrier width graded from 2 nm to 8 nm. Structure B3 has a fixed barrier width of 5 nm, with the quantum well width graded from 2 nm to 8 nm. The structures of the three samples are shown in Figure 3a.

Figure 3b shows the UV transmittance curves of the three sample groups. All three samples exhibit periodically oscillating interference fringes, indicating smooth material interfaces and good uniformity. At 260 nm, strong absorption is observed in all samples, corresponding to the Al_0.5_Ga_0.5_N buffer layer. The UV transmittance of all samples is approximately 10%, with periodically oscillating interference fringes. This phenomenon arises from light reflection and interference effects between the multi-layer interfaces of the AlN substrate, Al_0.5_Ga_0.5_N buffer layer, and AlGaN superlattice. The oscillations in Figure 3b are highly clear and regular, confirming excellent interface uniformity across all samples.

**Figure 4:**
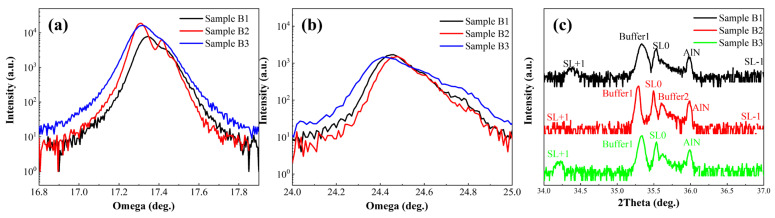
High-resolution X-ray diffraction (HR-XRD) patterns of the three structures. (**a**) (002)-plane rocking curves. (**b**) (102)-plane rocking curves. (**c**) (002)-plane 2θ-ω scans.

Figure 4a and Figure 4b present the rocking curves of the (002) and (102) planes for the three sample groups, respectively. The crystalline quality of the materials can be evaluated from the full width at half maximum (FWHM) of these curves. Given that the superlattice structure is very thin (~70 nm), the FWHM of the rocking curves primarily reflects the dislocation density inherited from the growth of the buffer layer. As shown in Table 1, Sample B2 exhibits a smaller FWHM in its rocking curves and lower dislocation density—particularly a significant reduction in screw dislocation density—indicating that the graded-barrier superlattice with a fixed well width demonstrates significantly better crystalline quality than the other two superlattice structures, with fewer crystal defects and improved crystallinity. The underlying reason is that the other two superlattice structures, which fix the barrier layer thickness during growth, exhibit growth behavior along the (002) direction analogous to that of a material with decreasing or constant Al composition. In contrast, the structure with a fixed well width shows growth along the (002) direction similar to that of a material with a smoothly decreasing Al composition. This smoother compositional variation effectively mitigates lattice mismatch stress, reducing the generation and propagation of dislocations.

Figure 4c displays the high-resolution X-ray diffraction (HRXRD) 2θ-ω scan patterns of the three sample groups. The diffraction peaks corresponding to the buffer layer and superlattice satellite peaks are clearly visible, with specific peak positions listed in Table 2. Compared to Samples B2 and B3, Sample B1 exhibits more distinct superlattice satellite peaks. This is attributed to the consistent thickness of the well and barrier layers in B1, resulting in uniform superlattice period thickness. Since superlattice diffraction follows the Bragg equation, the period thickness strongly influences the Bragg angle of the satellite peaks. Consequently, the satellite peak signals in Samples B2 and B3 are less pronounced.

**Figure 5:**
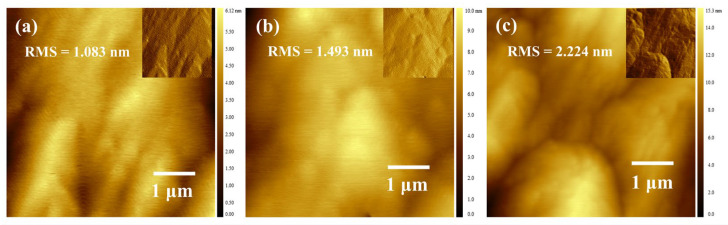
(**a**–**c**) Atomic Force Microscopy (AFM) images of the three structures (insets display shaded data transformations of the original images to enhance visualization of the steps).

Figure 5a–c present the AFM surface morphology images of the three sample groups. The insets in Figure 5 show shaded-processed AFM height maps, which clearly reveal the surface steps. All three samples exhibit distinct step features on their surfaces. Notably, the roughness of Samples B1 and B2 is lower than that of Sample B3. This discrepancy arises from the superlattice well-barrier structure; the surfaces of the samples correspond to a low-Al-composition AlGaN layer. Specifically, Samples B1 and B2 have a 5-nm-thick low-Al-composition AlGaN layer on their surfaces, whereas Sample B3 only has a 2-nm-thick layer. When the Al composition is low, the material grows in a 2D step-flow mode. As the Al composition increases, fewer atoms can reach the step edges, leading to a rougher surface and a higher RMS (root mean square) roughness. Although Sample B2 shares the same surface AlGaN layer thickness as Sample B1, its more varied periodic structure prior to surface growth results in a relatively poor surface morphology.

**Figure 6:**
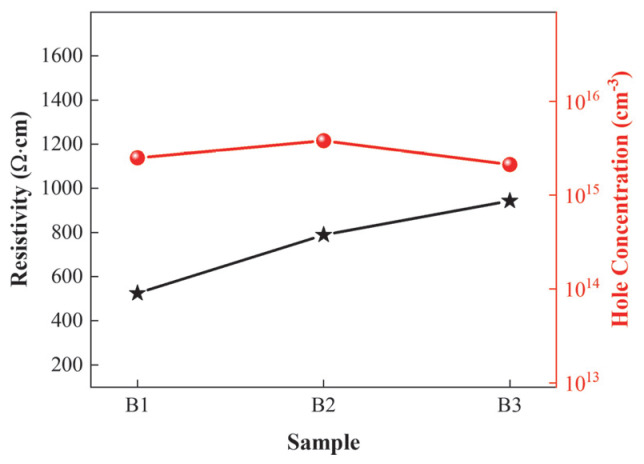
Hole concentrations and resistivities of the three superlattice structures.

Hall measurements were performed on the three sample groups, and the test results are shown in Figure 6. Sample B3 exhibits the poorest hole concentration and resistivity, which is attributed to the use of a graded well layer thickness in Sample B3. In the superlattice structure, the acceptors in the barrier layer are activated by capturing free electrons from the valence band maximum of the well layer, thereby reducing the acceptor activation energy. The thin well layer thickness in B3 reduces the number of activated acceptors, leading to low hole concentration and high resistivity. In contrast, Sample B2 has a higher hole concentration than Sample B1 but a lower resistivity than Sample B1. The reason is that Sample B2 maintains a fixed well layer thickness and employs a graded barrier layer thickness. Although this configuration contributes to increasing the hole concentration, the presence of thick barrier layers makes it difficult for holes to tunnel through and subjects them to greater scattering, thereby increasing resistivity.

**Figure 7:**
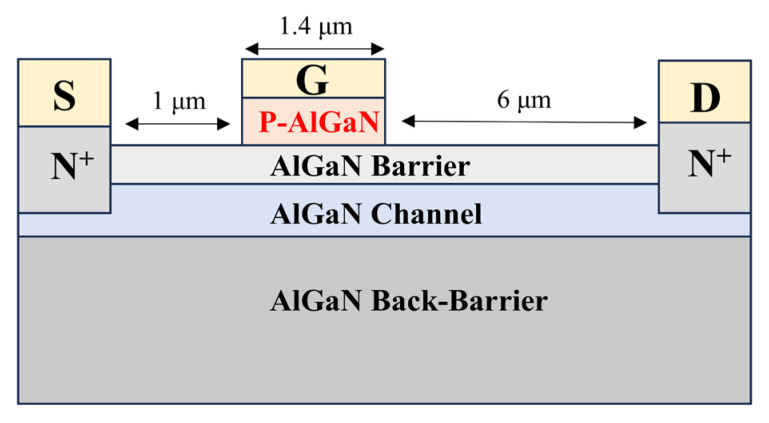
Schematic diagram of the enhancement-mode HEMT device structure.

Through simulation and experiments, we achieved high-efficiency p-type doping in AlGaN superlattice structures. Compared to p-GaN, p-AlGaN enables band structure design through Al composition tuning, thereby fabricating devices that meet the required specifications. By tuning the material under the gate, precise tuning of the HEMT threshold voltage can be achieved. In conventional AlGaN materials, the Mg acceptor activation energy increases from 170 eV in GaN to 630 eV in AlN as the Al composition increases. However, by adopting a superlattice structure, the acceptor activation energy can be significantly reduced, greatly improving the p-type doping efficiency of AlGaN materials. We simulated enhancement-mode HEMT devices using Silvaco TCAD software, with all devices being identical except for the p-type structure under the gate. The structure is shown in Figure 7.

Conventional HEMT devices typically use C-doped GaN as the buffer layer, GaN as the channel, and low-Al-composition AlGaN as the barrier layer. Their advantages include ease of material growth and simplicity of subsequent etching processes. However, this structure exhibits poor voltage withstand capability. To enhance the breakdown voltage of the devices, researchers have proposed using low-Al-composition AlGaN as the channel and high-Al-composition AlGaN as the barrier or back barrier. Although this structure offers better voltage withstand capability, using p-GaN as the material under the gate introduces a stronger polarization electric field, making it difficult to turn off the device. In this scenario, we need to introduce P-AlGaN with an Al composition close to that of the barrier layer as the gate control material to reduce the impact of polarization on the device switching capability.

**Figure 8:**
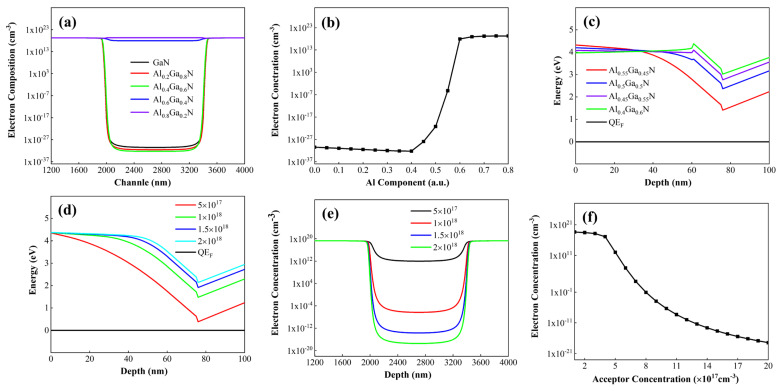
(**a**) Depletion effects of p-AlGaN with varying Al compositions under the gate on electrons in the channel. (**b**) Influence of Al composition in p-AlGaN material under the gate on electron concentration within the gate-channel region. (**c**) Effects of p-AlGaN with varying Al compositions under the gate on energy band structures in the gate region (**d**) Effects of p-AlGaN with varying doping concentrations under the gate on energy band structures in the gate region. (**e**) Influence of acceptor concentration in p-AlGaN material under the gate on carrier distribution in the gate-channel region, (**f**) Variation of electron concentration in the channel region under the gate with different acceptor doping concentrations.

In the device structure shown in Figure 7, the gate-source spacing is 1 μm, the gate-drain spacing is 6 μm, and the gate length is 1.4 μm. All structures have a p-type layer thickness of 60 nm, with the p-type layers being p-(Al)GaN and p-AlGaN superlattice, respectively. For simplicity, the enhancement-mode HEMT using p-(Al)GaN as the material under the gate will be referred to as the “conventional structure device,” and the one using p-AlGaN superlattice under the gate will be referred to as the “superlattice structure device” in the following text. When different Al compositions of AlGaN materials are used as the gate-under material in the conventional device structure, and the p-type region doping concentration is uniformly 1 × 10^18^ cm^−3^, the electron concentration in the channel region is shown in Figure 8.

Figure 8a illustrates the electron concentration distribution in the channel region of the conventional structure device under zero gate voltage. When the Al composition is zero, it corresponds to p-GaN. As observed in Figure 8a, with increasing Al composition in the p-AlGaN layer, the electron concentration at the gate-under channel first decreases and then increases. This is because as the Al composition in p-AlGaN increases, the p-type doping efficiency decreases, reducing the hole concentration and weakening the depletion effect on the two-dimensional electron gas (2DEG) in the channel.

When the Al composition of the p-type layer increases to match that of the channel layer, the polarization electric field reaches its maximum under the combined stress at the upper and lower interfaces of the barrier layer. When the Al composition of the p-type layer exceeds that of the channel layer, the tensile stress on the barrier layer decreases, weakening the polarization effect, thereby reducing the gate’s control over the underlying channel and increasing the electron concentration. If the Al composition of the p-type layer exceeds that of the barrier layer, the barrier layer is subjected to compressive stress from the p-type layer, generating more positive polarization charges at the interface between the barrier and channel layers, which in turn increases the electron concentration in the channel.

From Figure 8b, it can be seen that to control the channel switching via the gate, the Al composition of the p-AlGaN material under the gate should not exceed that of the barrier layer. On the other hand, as the Al composition in the p-type layer increases, the polarization effect strengthens, leading to greater conduction band bending and bringing the conduction band minimum closer to the Fermi level, as shown in Figure 8c. Therefore, the turn-on voltage of the HEMT device can be precisely controlled by adjusting the Al composition of the p-AlGaN material.

As depicted in Figure 8d, adjusting the doping concentration of the p-AlGaN material changes the band structures of the gate-under material, barrier layer, and channel layer. With increasing doping concentration, the distance between the conduction band and the Fermi level gradually increases, indicating an increase in the threshold voltage. Figure 8e,f show the electron concentration distributions in the channel under different acceptor doping concentrations. The electron concentration under the gate decreases with increasing acceptor doping. Figure 8f shows the electron concentration at the gate-under channel under different acceptor doping concentrations. As the acceptor doping increases, the electron concentration decreases with a gradually reduced slope, indicating that the influence of acceptor doping concentration on the channel approaches its peak.

**Figure 9:**
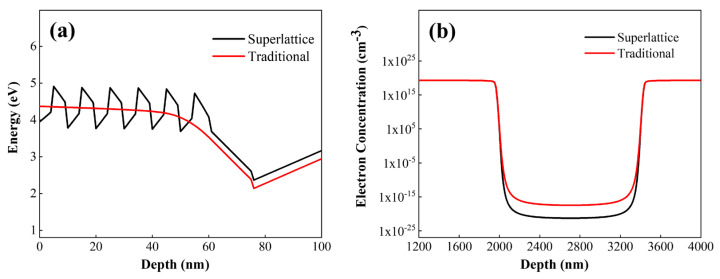
(**a**) Conduction band energies of conventional p-AlGaN structures vs. superlattice AlGaN structures under the gate. (**b**) Depletion effects of conventional p-AlGaN structures vs. superlattice AlGaN structures under the gate on electrons in the channel.

From Figure 8, it can be seen that the Al composition of the p-type layer and the acceptor doping concentration (hole concentration) collectively regulate the band structure. By controlling the electron concentration under the gate, the turn-on and turn-off of the channel can be modulated. Previous studies have shown that superlattice doping facilitates acceptor activation and helps increase the hole concentration. We replaced the conventional structure with a p-AlGaN superlattice structure, ensuring that the average Al composition of the p-type layer under the gate is 0.55, both the superlattice barrier and well thicknesses are 5 nm, and the acceptor doping concentration is 2 × 10^18^ cm^−3^. The results are shown in Figure 9.

Figure 9a presents the energy band diagrams of the p-type region under the gate, the barrier layer, and the channel layer. The two structures have the same Al composition in the p-type material under the gate, meaning the stress on the barrier layer is consistent, and the effects caused by piezoelectric polarization are consistent. However, when the superlattice is used as the p-type material under the gate, the higher hole concentration compared to the conventional structure leads to an upward shift of the conduction band, resulting in a larger threshold voltage and enhanced gate control capability. Figure 9b shows the electron concentration distribution in the channel region of the superlattice structure device. By using the superlattice structure as the p-type layer under the gate, compared with the conventional structure, the hole concentration is higher under the same doping concentration. More electrons in the channel are depleted, which helps increase the threshold voltage.

## 3. Conclusions

This work simulated the structures and performances of superlattices with different configurations using Silvaco TCAD. Three types of superlattices were grown via MOCVD, and their surface morphologies, crystalline qualities, and electrical properties were characterized. Finally, p-type doping was achieved in high-Al-content AlGaN materials. It proposed using p-AlGaN superlattices as the material under the gate of HEMT devices to achieve enhancement-mode devices. Simulations verified that the superior p-type doping capability of p-AlGaN superlattices facilitates achieving a larger threshold voltage.

## 4. Discussion

For traditional gate-under p-GaN and barrier-layer AlGaN, high-selectivity etching is achievable via gas chemistry optimization. For instance, Zhang et al. [33] reported that an inductively coupled plasma (ICP) etching process using BCl_3_/SF_6_ gas mixture achieved a selectivity of 41:1 for p-GaN over AlGaN, with the AlGaN surface remaining atomically smooth (RMS roughness = 0.428 nm) due to the formation of non-volatile AlFx etch byproducts that act as a self-limiting stop layer. In contrast, p-AlGaN superlattices—composed of alternating AlGaN layers with varying Al compositions—lack a distinct etch-stop interface. 

The use of p-AlGaN superlattices as the material under the gate is beneficial for devices requiring precise threshold voltage design. However, in practical processes, the gate-under p-AlGaN material often requires dry etching for fabrication. For conventional gate-under p-GaN and barrier-layer AlGaN, high-selectivity etching can be achieved by introducing an O_2_/Cl_2_ gas mixture, enabling the etch to stop on the barrier layer. For example, Yin et al. achieved a selectivity ratio of 33:1 and minimal damage to the AlGaN barrier [34]. When using p-AlGaN superlattices as the gate-under material, dry etching struggles to achieve high selectivity, and only time-controlled etching can be employed, resulting in poor surface morphology. Therefore, time-controlled etching needs to be combined with thermal annealing to form a relatively smooth surface.

## Figures and Tables

**Table 1:** Full width at half maximum (FWHM) of rocking curves and dislocation densities for (002) and (102) planes of the three configurations.

Sample	FWHM		Dislocation Density	
	(002)	(102)	ρ_screw_	ρ_edge_
B1	436	557	7.1 × 10^8^	1.2 × 10^9^
B2	406	446	6.1 × 10^8^	7.2 × 10^8^
B3	449	607	7.5 × 10^8^	2 × 10^9^

**Table 2:** Peak positions and structural information of the superlattice structures.

Sample	B1	B2	B3
SL-1	34.5	34.22	34.23
SL0	35.52	35.51	35.53
SL+1	36.35	36.6	36.64
Buffer 1	35.28	35.3	35.33
Buffer 2	35.7	35.72	35.74
AlN	35.98	35.98	35.98
Composition	0.54	0.55	0.55
Thickness	9.6	7.3	7.4

## Data Availability

All data that support the findings of this study are included within the article.
